# Translational Development and Application of (1→3)-β-d-Glucan for Diagnosis and Therapeutic Monitoring of Invasive Mycoses

**DOI:** 10.3390/ijms18061124

**Published:** 2017-05-24

**Authors:** Matthew W. McCarthy, Ruta Petraitiene, Thomas J. Walsh

**Affiliations:** 1 Division of General Internal Medicine, Weill Cornell Medicine of Cornell University, New York, NY 10065, USA; 2 Transplantation-Oncology Infectious Diseases Program, Division of Infectious Diseases, Department of Medicine, Weill Cornell Medicine of Cornell University, New York, NY 10065, USA; rop2016@med.cornell.edu; 3 Departments of Pediatrics, and Microbiology & Immunology, Weill Cornell Medicine, New York, NY 10065, USA; thw2003@med.cornell.edu

**Keywords:** antigenemia, pulmonary aspergillosis, ventriculitis, meningitis, candidiasis

## Abstract

Early diagnosis and prompt initiation of appropriate antimicrobial therapy are crucial steps in the management of patients with invasive fungal infections. However, the diagnosis of invasive mycoses remains a major challenge in clinical practice, because presenting symptoms may be subtle and non-invasive diagnostic assays often lack sensitivity and specificity. Diagnosis is often expressed on a scale of probability (proven, probable and possible) based on a constellation of imaging findings, microbiological tools and histopathology, as there is no stand-alone assay for diagnosis. Recent data suggest that the carbohydrate biomarker (1→3)-β-d-glucan may be useful in both the diagnosis and therapeutic monitoring of invasive fungal infections due to some yeasts, molds, and dimorphic fungi. In this paper, we review recent advances in the use of (1→3)-β-d-glucan to monitor clinical response to antifungal therapy and explore how this assay may be used in the future.

## 1. Introduction

The Fungitell assay (Associates of Cape Cod, Inc., East Falmouth, MA, USA) is a commercial test that detects (1→3)-β-d-glucan (BDG), which is an important component of the fungal cell wall [1,2]. The BDG assay can be used to diagnose some invasive fungal infections (IFIs) in humans and employs the same principle as serum endotoxin assays, measuring activation of Factor G through horseshoe crab substrates [3]. The output of the serum assay is based on spectrophotometer readings, in which optical density is converted to BDG concentrations, and the results are interpreted as negative (range < 60 pg/mL), indeterminate (60 pg/mL to 79 pg/mL), or positive (>80 pg/mL) [4]. Precise cut-offs to optimize performance of the assay to diagnose IFIs have not been defined and may be different based on the host and pathogen.

The BDG assay can be positive in patients with a variety of IFIs, including candidiasis, cryptococcosis, aspergillosis, and *Pneumocystis jirovecii* (formerly *P. carinii*) pneumonia but it is typically negative in patients with mucormycosis or fusariosis [5,6,7,8]. The role of serum BDG testing to diagnose IFIs has been well documented, but emerging data suggest that the assay may also have important clinical implications in other samples, including bronchoalveolar lavage (BAL) fluid and cerebrospinal (CSF) fluid [9,10,11]. In this paper, we review the role of BDG testing in serum, BAL, and CSF samples to detect IFIs and monitor clinical response to antifungal therapy and conclude with an exploration of future directions for the assay and ways in which it might be more effectively implemented into clinical practice.

## 2. (1→3)-β-d-Glucan (BDG) in Serum

Most clinical experience with BDG testing to monitor response to antifungal therapy involves serum. This includes testing for yeast and yeast-like fungi such as *Candida* spp. and *Pneumocystis jirovecii* as well as molds such as *Aspergillus* spp. Despite the substantial heterogeneity among different studies, the BDG assay has good accuracy for distinguishing patients with proven or probable IFIs from patients without invasive fungal infection and in some cases can be used to monitor response to treatment [12,13,14,15,16]. In this section, we review seminal studies and recent advances in serum BDG testing to monitor response to antifungal therapy.

### 2.1. Candidiasis

The incidence of invasive candidiasis (IC) has been increasing over the past thirty years and is a common cause of nosocomial bloodstream infections [17,18]. The gold standard for the diagnosis of candidemia is a positive blood culture, but this method is plagued by poor sensitivity, and the serum BDG assay can be a useful adjunct, especially in patients with intraabdominal candidiasis [19]. Results from a single center, observational study suggest that falling BDG levels correlate with clinical outcome in patients with IC, but this work was limited by small sample size and the observational nature of the investigation [20].

The role of BDG testing to monitor response to therapy in patients with IC was also evaluated by Jaijakul and colleagues, who plotted serial BDG levels collected over time from 203 patients with proven IC during treatment with the echinocandin anidulafungin [21]. Using this charting method, the authors correlated a negative slope in BDG levels from patients with a favorable treatment outcome (PPV of 90%) and a positive slope following treatment failure (NPV of 90%). These findings suggest that for patients with IC, trending of BDG values can be a useful prognostic marker for response to antifungal treatment.

These findings should be met with a note of caution, however, because the study had limited data on potential confounding factors such as catheter removal, previous antifungal use, treatment with albumin or immunoglobulin, renal insufficiency, sponges or gauze exposure, or hemodialysis, all of which may affect BDG levels [13,22]. The study was not designed or powered to evaluate the usefulness of BDG level as a survival predictor and the presence or absence of serum BDG should not be used to guide cessation of therapy. It is crucial for clinicians to know that the BDG assays should always be interpreted in the context of clinical, radiographic, and microbiological findings [23].

### 2.2. Pneumocystis jirovecii Pneumonia

*Pneumocystis jirovecii* pneumonia (PJP) classically develops in immunocompromised hosts with acquired immunodeficiency syndrome (AIDS), prolonged corticosteroid exposure, or other disorders of T-cell function [24,25,26,27]. Patients typically present with dyspnea, non-productive cough, and fever in the setting of diffuse ground glass opacities on chest radiograph [27,28]. The preferred diagnostic specimens for detection of *P. jirovecii* are BAL fluid or biopsy, but obtaining this material may not be possible in some patients [29,30]. The utility of the serum BDG assay to diagnose PJP was illustrated in a retrospective case-control study of 295 patients with suspected infection [31]. The BDG assay had a sensitivity of 92 percent and a specificity of 86 percent for detecting PJP, suggesting that it might also have some role in the therapeutic monitoring.

A retrospective study investigated whether consecutive serum BDG measurements could be used to assess treatment response in patients with PJP [32]. Analysis of sera from 18 patients during PJP therapy reveals that decreasing BDG-levels strongly correlated with a response to antifungal therapy. However, increasing BDG-levels were associated with treatment failure or fatal outcome in less than half (44%) of patients, indicating that serum BDG testing might be used to confirm treatment success but seem to be of limited value for the identification of treatment failure.

### 2.3. Aspergillosis

Invasive aspergillosis (IA) most frequently occurs in the lungs or sinuses of immunocompromised hosts after inhalation of conidia [33,34,35]. Typical risk factors include severe and prolonged neutropenia, high-dose corticosteroid therapy, human immunodeficiency virus (HIV) infection, solid organ transplant, hematopoietic stem cell transplant, and chronic granulomatous disease [36,37,38,39]. IA can also occur patients with less severe immune impairment in the intensive care unit setting, particularly those with underlying chronic obstructive pulmonary disease COPD [40,41].

*Aspergillus* spp. are ubiquitous in nature and are frequently inhaled into the human airways [42]. Thus, isolation of the organism from the airways does not necessarily indicate disease [43]. The diagnosis of IA can be challenging in clinical practice and is based upon both isolation of the organism (or biomarkers of the organism such as BDG) and the probability that it is the cause of disease [13,44]. The latter is a function of the host's immune status as well as presenting symptoms, which can be subtle and non-specific [45]. Demonstration of hyphal elements invading tissues (from biopsy of any affected site, such as the lung or skin) represents a proven diagnosis [46,47].

In some cases, however, tissue sampling is infeasible due to host factors such as thrombocytopenia, which may preclude biopsy. In this setting, detection of circulating surrogate biomarkers of IA such as BDG or galacatomannan (GM) may be useful not only for diagnosing disease but also for assessing the effectiveness of therapy [48]. This has been demonstrated for GM antigenemia, since declining levels of GM have been found in patients responding to antifungal treatment while rising GM antigenemia has been associated with treatment failure [49,50]. The role of serum BDG testing in the therapeutic monitoring of patients with IA was first established by Pazos and colleagues in a retrospective evaluation of 40 high risk, neutropenic adult patients [51].

In this study, five proven IA cases, three probable IA cases, and three possible IA were identified. All five patients with proven IA (100%), two (66%) of three patients with probable IA, and one (33%) of three patients with possible IA tested positive for serum BDG. In patients with proven IA, BDG levels showed a constant rise before clinical and microbiological evidence of IA existed and then decreased and ultimately became negative if the patient responded to antifungal treatment. Patients not responding to antifungal therapy did not show a decrease in the levels of BDG, suggesting that BDG antigenemia may be useful in predicting therapeutic outcomes in patients with IA.

Other groups have also shown that trending BDG antigenemia is a useful noninvasive method for monitoring response to antifungal therapy in patients with IA [52,53]. Results presented in this study showed, for the first time, that monitoring BDG antigenemia is useful in predicting the therapeutic outcome of patients with IA. Decreasing levels of BDG were observed in patients who recovered from IA, while patients not responding to antifungal treatment showed a continuous rise in serum BDG levels.

## 3. BDG in Bronchoalveolar Lavage (BAL)

There is limited data on the application of the BDG assay using BAL fluids [13,54,55]. One prospective, non-interventional multicenter study evaluated the utility of BDG in BAL fluids for early diagnosis of invasive fungal infections in solid organ transplant recipients found that the assay had a low positive predictive value (PPV) for BAL samples [56]. However, other work suggests serial BAL sampling may have an important role in diagnosis and response to antifungal therapy, which will be reviewed below.

### 3.1. Candida Pneumonia

Pneumonia due to *Candida* spp. is exceedingly rare [57]. The bronchoalveolar lavage (BAL) is a widely accepted diagnostic tool to confirm the diagnosis of pneumonia and to identify the nature of the associated pathogen, but its role in the diagnosis of *Candida* pneumonia is uncertain [58]. Similarly the role of BAL BDG sampling to diagnose and monitor this condition has not been elucidated. Recently, a prospective, observational study enrolled immunocompromised, critically ill, and ventilated patients with suspected fungal pneumonia in mixed intensive care units and obtained measurements of BDG BAL levels in these patients [59].

Thirty-one patients completed the study and were categorized into non-*Candida* pneumonia/non-candidemia (*n* = 18), suspected *Candida* pneumonia (*n* = 9), and non-*Candida* pneumonia/candidemia groups (*n* = 4). Levels of BDG in BAL were highest in suspected *Candida* pneumonia, while the serum BDG levels were highest in non-*Candida* pneumonia/candidemia. For the detection of suspected *Candida* pneumonia, the predictive performance (sensitivity/specificity/d-glucan cutoff (pg/mL) of BDG in BAL fluid was 89%/86%/130, suggesting this assay could serve as an adjunctive modality to detect suspected *Candida* pneumonia. Further work is needed to evaluate its potential role in monitoring response to antifungal therapy.

### 3.2. Invasive Pulmonary Aspergillosis

The BDG assay is widely appreciated to be a reliable adjunctive diagnostic modality in the diagnosis of invasive pulmonary aspergillosis (IPA) but its role in monitoring response to antifungal therapy remains an area of active inquiry [60,61,62,63]. Petraitiene and colleagues have used a neutropenic rabbit model and a nonneutropenic cyclosporine-methylprednisolone immunosuppressed rabbit model of experimental IPA to evaluate the burden of BDG in BAL fluid [64]. Neutropenia in rabbits was established with intravenous administration of cytarabine (Ara-C) beginning one day before *Aspergillus* inoculation. Antifungal therapy with intravenous liposomal amphotericin B (LAMB) at 5 mg/kg/day was initiated 24 h after inoculation and continued throughout the course of the experiments for 12 days.

Levels of BDG in BAL fluid were substantially higher in rabbits with cytarabine-induced neutropenia than in nonneutropenic animals with cyclosporine-methylprednisolone induced immunosuppression. However, there were no significant differences among the mean BDG levels in BAL fluid. There was a lack of effect of treatment with LAMB on BDG BAL levels, which argues against a role in therapeutic monitoring. This could be due to lack of pulmonary clearance of viable and nonviable cell wall elements and underscores that further work is necessary to elucidate the potential role, if any, that serial BDG BAL measurements could have in monitoring response to therapy in IPA [65].

## 4. BDG in Cerebrospinal Fluid (CSF)

The BDG assay have been approved by the United State Food and Drug Administration (FDA) for serological diagnosis of invasive fungal disease, but BDG testing is not approved for cerebrospinal fluid (CSF), and the appropriate cutoff value remains controversial [9,66,67,68]. Recent data suggest that the BDG assay may be useful for the early identification of some invasive mycoses and for monitoring antifungal therapy efficacy [69]. Below, we review recent advances in the application of the BDG CSF assay.

### 4.1. Hematogenous Candida Meningoencephalitis

Hematogenous *Candida* meningoencephalitis (HCME) is a potentially-devastating infection in pediatric patients that can cause seizures, cortical blindness, intraventricular hemorrhage and neurocognitive impairment [70,71,72]. As with other fungal infections of the central nervous system, diagnosis can be difficult and recurrence after completion of antifungal therapy is not uncommon [73,74]. A laboratory animal model of HCME has demonstrated excellent sensitivity of BDG in CSF, suggesting that this assay could be used to monitor therapeutic response in human patients [70].

A multicenter retrospective study was conducted that included chart reviews of pediatric subjects, aged birth to 18 years, who had neurologic symptoms, who had a working diagnosis of HCME (or CNS aspergillosis), and whose CSF was evaluated for the detection of BDG [75]. Seven patients were identified with *Candida* spp. and all had BDG detectable in their CSF. Among patients who completed antifungal therapy, all had elevated CSF BDG levels decreased to less than 31 pg/mL, suggesting the assay could play an important role in monitoring response to therapy. However, the study size was small and CSF sampling was limited in some patients due to prematurity. Nonetheless, this work lays the foundation for prospective multicenter studies to evaluate the role of BDG sampling in pediatric HCME.

### 4.2. Cryptococcal Meningitis

The role of BDG testing in cryptococcal meningitis has been controversial. BDG is expressed in the cell walls of many medically important fungi, but it was once believed that *Crytococcus neoformans* was not one of them [76]. However, in a study of HIV-infected Ugandan and South African adults with suspected meningitis, high concentrations of BDG were detected in CSF of patients with cryptococcal meningitis [8]. This was the first study to systematically assess CSF BDG levels in a cohort of patients with cryptococcal meningitis, and showed 89% sensitivity and 85% specificity from 117 diagnostic CSF specimens. In this group, detectable levels of BDG in CSF were found to correlate with quantitative fungal cultures and high CSF BDG levels (>500 pg/mL) were associated with a three times higher risk of 10-week mortality.

Although CSF BDG levels do not have adequate sensitivity or specificity to make this assay the preferred cryptococcal diagnostic test, positive results should warrant further diagnostic testing, especially in high-risk, immunocompromised patients [77]. Levels of BDG in CSF were found to rapidly decline with the start of antifungal therapy, falling by approximately 50% after 4 days. This is in contrast to cryptococcal antigen titers, which may continue to persist long after the completion of antifungal therapy, suggesting that CSF BDG could one day be used as a prognostic indicator for mortality and as a way to monitor response to treatment [78].

### 4.3. Aspergillus Ventriculitis

*Aspergillus* ventriculitis is a rare but potentially devastating form of invasive aspergillosis of the central nervous system that usually presents with parenchymal brain lesions (abscesses, hemorrhagic/ischemic lesions) in the setting of altered mental status and/or focal neurologic deficitis [79,80,81]. Little is known about the clinical management of this disease, including the potential role of serial monitoring of CSF BDG to monitor response to antifungal therapy.

A recent case report evaluated the utility of the CSF BDG assay in a 16-year-old boy who was diagnosed with germinoma of the pituitary and pineal gland and subsequently developed *Aspergillus* ventriculitis [82]. In this patient, levels of CSF BDG were elevated in the initial detection of *Aspergillus* ventriculitis above normal CSF levels of <31 pg/mL by >50-fold. CSF was sampled once every two to six weeks, and new onset of recurrent headache, altered mental status, or seizures was associated with a rise of CSF BDG levels in several instances. During the two-year period, CSF BDG decreased from a maximum level of 1575 to <31 pg/mL and CSF white blood cell count decreased from a peak of 1233 cells/μL to 0 cells/μL, in association with resolution of fever and headache. Interestingly, serum BDG levels were within normal limits and thus could not be used as surrogates for measurement of CNS BDG levels.

The treatment course was concluded after two consecutive negative BDG levels in CSF over a 4-week period. This normalization coincided with ultimate resolution of most lesions on MRI scan and the patient has now been in remission of his pineal/pituitary germinoma and *Aspergillus* ventriculitis for more than five years, indicating that CSF BDG monitoring can play an important role in the management of this condition.

### 4.4. Exserohilum Rostratum Meningitis

The 2012 nationwide outbreak of *Exserohilum rostratum* meningitis in the United States that was linked to contaminated epidural injections of methylprednisolone acetate brought renewed attention to the challenges of diagnosing and treating dematiaceous mold infections of the central nervous system [83,84]. *E. rostratum* is an opportunistic mold that until 2012 was a rare cause of disease in humans. In September of that year, health officials began to respond to a multistate outbreak of fungal meningitis traceable to three lots of preservative-free methylprednisolone from one compounding pharmacy in Massachusetts [85,86]. This outbreak resulted in hundreds of cases of CNS infection and scores of deaths across the United States [85,87].

Isolation of the causative organism was considered the gold standard for diagnosis, but assays for BDG in CSF were developed to potentially serve as adjunctive diagnostic tools [88]. In one study, the sensitivity and specificity of BDG testing was evaluated by testing 41 CSF specimens from confirmed cases of *E. rostratum* meningitis and 66 negative control specimens [89]. A cutoff value of 138 pg/mL provided 100% sensitivity and 98% specificity for the diagnosis of fungal meningitis in this outbreak and most patients had experienced a decline in CSF BDG displayed clinical improvement. Conversely, three patients with continually elevated CSF BDG had poor clinical outcomes (stroke, relapse of meningitis, or development of new disease). The data suggest that serial assessment of CSF BDG could be used monitor clinical response and may provide useful adjunctive data for the management of outbreak-associated meningitis.

### 4.5. Coccidioidal Meningitis

Coccidioidal meningitis (CM) is a rare but deadly complication of coccidioidomycosis and has a documented mortality if untreated of 90% in 1 year and 100% in 2 years [90,91,92]. The diagnosis of CM is an ongoing challenge in clinical practice owing to its infrequency, non-specific presenting symptoms, and/or a delay in the positivity of a CSF culture or antibody [93,94,95]. In this setting, CSF BDG has emerged as a potential adjunctive diagnostic modality.

In one study, 37 CSF specimens were evaluated for BDG; 26 patients had confirmed CM and 11 patients had suspected microbial meningitis without a fungal diagnosis [66]. For patients with CM, BDG levels in CSF specimens ranged from 18 to 3300 pg/mL while in controls it ranged from <3.9 to 103 pg/mL. Diagnostic performance was determined using a 31-pg/mL cutoff; sensitivity was 96%, specificity was 82%, positive and negative predictive values were 93% and 90%, respectively. These findings suggest that for patients with suspected CM, the CSF BDG assay may be more sensitive than coccidioidal CSF antibody for diagnosis. This study lays the conceptual framework for further research involving CSF BDG in patients with suspected or confirmed CM, including studies evaluating the role of serial CSF BDG sampling to monitor response to antifungal therapy.

## 5. Conclusions and Future Directions

An emerging body of literature suggests that for patients with IFIs, the BDG assay may have an important role in monitoring response to antifungal therapy. The cost per BDG assay can vary (depending on contracts, the performing laboratory, and specimen type) but typically ranges between $100 and $200 [22]. The economic burden of IFIs can be substantial, and serial BDG testing has the potential to significantly decrease patient cost if it is used in the appropriate setting [96,97,98]. Repeatedly positive BDG results and/or increasing BDG levels may prompt sooner initiation of antifungal therapy or may lead to a change in treatment. Conversely, decreasing BDG levels may lead to appropriate cessation of antifungal therapy, which may prevent treatment-associated complications as well as the emergence of fungal resistance.

Conceptual cost-savings must be borne out in clinical trials, however, and prospective studies evaluating the economic impact of serial BDG monitoring are needed to demonstrate that this approach is cost-effective in targeted, high-risk patients. We believe that in the coming years, the BDG testing will play an important role in therapeutic monitoring in selected patients with IFIs and high quality data will be crucial to properly implement this important assay in clinical practice.
